# Post-Processing of FDM 3D-Printed Polylactic Acid Parts by Laser Beam Cutting

**DOI:** 10.3390/polym12030550

**Published:** 2020-03-03

**Authors:** Mahmoud Moradi, Mojtaba Karami Moghadam, Mahmoud Shamsborhan, Mahdi Bodaghi, Hamid Falavandi

**Affiliations:** 1 Department of Mechanical Engineering, Faculty of Engineering, Malayer University, Malayer P.O. Box 65719-95863, Iran; moradi.malayeru@gmail.com (M.M.); mojtaba.kmoghadam1991@gmail.com (M.K.M.);; 2 Laser Materials Processing Research Centre, Malayer University, Malayer P.O. Box 65719-95863, Iran; 3 Department of Engineering, Mahabad Branch, Islamic Azad University, Mahabad P.O. Box 59135-433, Iran; m.shamsborhan@gmail.com; 4 Department of Mechanical Engineering, University of Zakho, Zakho P.O. Box 12, Kurdistan Region, Iraq; 5 Department of Engineering, School of Science and Technology, Nottingham Trent University, Nottingham NG11 8NS, UK

**Keywords:** post-processing, additive manufacturing, laser cutting, fused deposition modeling, 3D printing, design of experiments

## Abstract

In this paper, the post-processing of 3D-printed poly lactic acid (PLA) parts is investigated. Workpieces are manufactured by fused deposition modeling (FDM) 3D printing, while they may have defects in some areas such as edges. A post-processing is introduced here for 3D-printed samples by low power CO_2_ laser. The thickness of the FDM samples are 3.2 mm and printed by optimum conditions. Effects of process parameters such as focal plane position (−3.2–3.2 mm), laser power (20–40 W), and laser cutting speed (1–13 mm/s) are examined based on the design of experiments (DOE). Geometrical features of the kerf; top and bottom kerf; taper; ratio of top to the bottom kerf are considered as output responses. An analysis of the experimental results by statistical software is conducted to survey the effects of process parameters and to obtain regression equations. By optimizing of the laser cutting process; an appropriate kerf quality is obtained and also optimum input parameters are suggested. Experimental verification tests show a good agreement between empirical results and statistical predictions. The best optimum sample with 1.19 mm/s cutting speed, 36.49 W power and 0.53 mm focal plane position shows excellent physical features after the laser cutting process when 276.9 μm top and 261.5 μm bottom kerf width is cut by laser.

## 1. Introduction

Laser material processing (LMP) has been implemented as a useful method in many industrial applications. For instance, high accuracy and quick operation are provided by the laser material processes [1]. Many of workpieces manufactured by traditional and non-traditional methods need post-processing for improving the quality of the processes [2]. LMP methods are beneficial for different industrial applications. For example, laser welding, laser surface hardening, laser drilling, laser additive manufacturing, laser engraving, laser forming, laser machining, and laser cutting are some of the useful applications of the laser technologies [3,4,5,6,7,8,9,10,11]. In the well known laser cutting process, by focusing the laser beam on a particular point, the material is cut off by the laser. First it melts and then evaporates [12,13,14]. The mixture of many processes is extremely interesting, and the main aims of many huge factories reach to a superb quality, whereas the time and money are saved. Currently, additive manufacturing (AM) is becoming more and more extensive in various areas such as architecture, medical and industrial fields [15]. In the AM methods, a 3D workpiece is produced by adding layers of substance. The AM processes are typically more sustainable than traditional manufacturing processes, because they waste less energy and material [16,17,18,19,20]. Polylactic acid (PLA) has had the second largest amount of utilization of all bioplastics in the world [21,22,23]. Recycling and biodegradability of PLAs has increased the importance of this material in the new technological world, where they do not have any disadvantages for the environment [24,25,26,27,28,29,30,31]. Furthermore, corn starch is one of the richest source of PLA. These materials, after being released into nature, are degradable and capable of being eventually degraded. In other words, they are biodegradable and biocompatible [32]. The viscosity of polymer melts is mainly determined by chemical structure, molecular weight, as well as temperature and shear rate in the course of processing and PLA itself could be either amorphous or semi-crystalline based on its chirality [33].

Many research works have investigated the laser cutting process. The laser cutting process of fiber glass sheets with changing inputs of laser cutting process was investigated by Choudhury et al. [34]. The material thickness, speed of CNC table and nozzle diameter on kerf quality were studied. Caiazzo et al. [35] examined the laser cutting process of different polymeric plastics such as PC, PE and PP by a high-power CW laser. The range of laser variation (laser power and laser cutting speed) showed that the laser power is more effective when the cutting speed is low. Additionally, the thickness of samples had an effective role on laser inputs. Zhou et al. [36] investigated the relationship between the theoretical and experimental study of laser cutting by CO_2_ laser. In the following, some studies which have used this method for the laser cutting process are discussed. The design of experiments (DOE) is a well known approach for laser material processing and many researchers in the last decade have conducted many investigations on it. By determining input and output variation of the process and also specifying certain experiments, it can reach the optimization condition parameters for each process. Davim et al. [37] tried to improve the cutting quality of poly methyl methacrylate (PMMA) edges by CO_2_ laser. The conclusion depicted that the heat affected zone (HAZ) dimensions were between 0.12 and 0.37 mm, without bore. Additionally, the roughness of the surface was very low. In highly advanced industries, nanocomposites have many applications, whereas their processing is a tough function. The addition of carbon nanotubes to plastics for improving mechanical properties have many effects on the post-production processes of these composites. For example, Ayob Karimzad et al. [38] used response surface methods (RSMs) in the laser cutting process of nanocomposites containing carbon nanotubes. Inputs variations such as focal plan position (FPP), laser power and laser cutting speed were selected. Results showed that the least dimension of the kerf width was 1.5 %. Eltawahni et al. [39] studied a Box–Behnken design and laser cutting process dependency. The conclusion showed that the laser inputs (laser power, cutting speed and FPP) had good significant conditions in the ANOVA tables for all the outputs results. 

The post-processing of additive manufacturing is sometimes essential for many industrial products and more precise applications. Based on the relevant literature, investigations on the laser cutting of 3D-printed parts by fused deposition modeling (FDM) is rare. Therefore, pioneer investigations should be carried out, in order to analyze possible advantages of the laser cutting on the printed parts. In this paper, the effects of laser beam on top and bottom kerf width, ratio of the top kerf to bottom kerf and kerf taper in the CO_2_ laser cutting of PLA sheets 3D-printed by FDM are investigated. The optimization of laser cutting is investigated in order to achieve the best geometrical objects, whereas the quality of kerf are preserved. On account of the low dimensional accuracy of the components produced in the laminate process, post-machining processes are required. Consequently, the laser is used to improve the dimensional accuracy.

## 2. Experimental Design and Methodology

In this study, the RSM is used to output variables (responses) [40,41,42]. The purpose of this method is to find a logical mathematical relationship between input and output variables. When all autonomous variables can be measured during a study, the response surface is to be asserted as a function by Equation (1) [43,44,45,46]:

Y= f(x_1_, x_2_, x_3_, …, x_k_) (1)

where “k” is the autonomous changeable number (independent variables). A quadratic polynomial function is assumed in the RSM with regard to the output responses as [47,48,49,50]:

(2) $$y=\beta_{0}+\overset{k}{\underset{i=1}{\sum }}\beta_{i}x_{i}+\overset{k}{\underset{i=1}{\sum }}\beta_{ii}x_{i}^{2}+\underset{i}{\sum }\underset{j}{\sum }\beta_{ij}x_{i}x_{j}+\varepsilon$$

β_i_ in this equation is a linear coefficient, β is a constant term, the term β_ij_ is an interaction coefficient, β_ii_ is a coefficient of quadratic and ε is the error term. Three variable laser parameters which have been taken into account in this experimental work are mentioned in Table 1.

**Figure 1:**
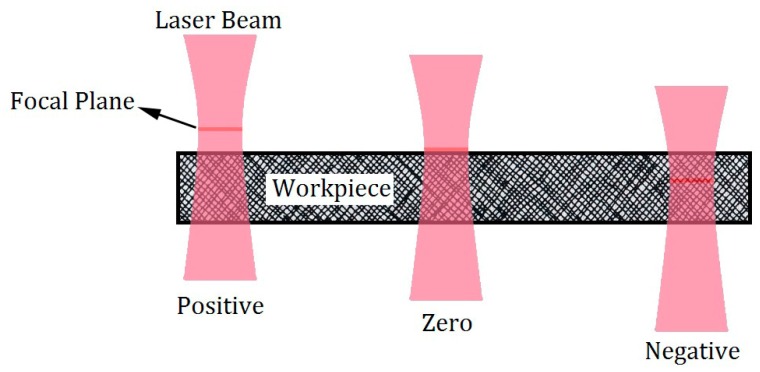
Alteration of figure plan position (FPP) on the sheet.

According to Figure 1, FPP has 3 positions (positive, zero and negative position) at workpieces. In particular, when FPP is discussed in this research, the focal length is in the position of the workpiece, which is precisely the FPP on top of the sheet at zero position.

Table 2 shows the input variables as well as the measured values for output responses of 17 experiments. FPP, laser cutting speed and laser power are selected as input laser parameters for laser cutting process in this study. Additionally, the top and the bottom kerf width, ratio of the top kerf to bottom kerf width and taper are considered as output experimental parameters.

## 3. Experimental Work

### 3.1. Polylactic Acid Sheet Fabricated by 3D Printing

**Figure 2:**
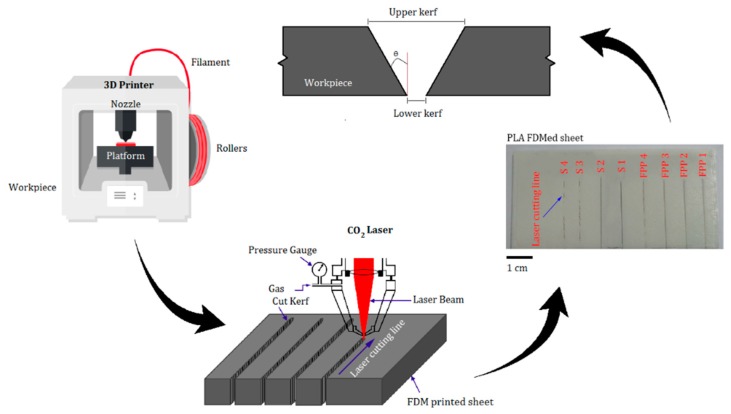
A schematic of the experimental process.

Figure 2 shows the process from fabricating PLA samples by FDM 3D printing to the laser cutting process by CO_2_ low power laser. After printing the sheets by FDM process, the laser cutting process is performed on the CNC table.

In order to produce PLA sheets, 3D printing is utilized by FDM technology. The simplified software is used to set parameters for manufacturing 3D samples, see Figure 2 and Table 3. In the 3D printer, extruder temperature (230 °C), infill percentage (16.86 %) and layer thickness (0.23 mm) are selected as constant parameters by optimum settings [29]. A bioactive and biodegradable PLA sheet with dimensions of 10 × 5 cm and a thickness of 3.2 mm is fabricated. Table 4 shows properties of the PLA.

### 3.2. Laser Cutting Process

**Figure 3:**
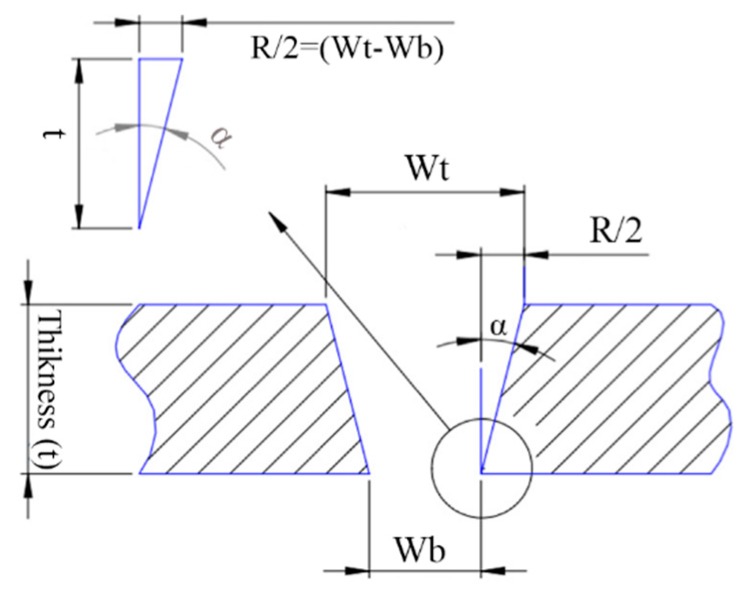
Cross-section of the top and bottom kerf.

**Figure 4:**
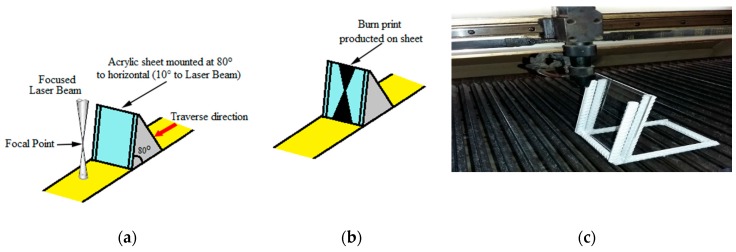
Determination of the laser beam focal point: (**a**) before passing through laser, (**b**) effect of the laser beam on the acrylic sheet that identifies the location of the focal point, (**c**) FPP determining with the CO_2_ laser.

In this present study, 60 Watts of CO_2_ is examined for the laser cutting process on the PLA sheets. The geometric characteristics of the cut are the width of the uppercut, the width of the lower cut, taper, and the ratio of the width of the incision to the uppercut to the lower cut width, as shown in Figure 3. The geometric characteristics are shown in the transverse section of the cutting kerf. The geometric features (such as top and bottom kerf width) were measured by the ImageJ software. Equation (3) defines the tapering angle as depicted in Figure 3:

(3) $$\alpha =\tan^{-1}\frac{w_{t}-w_{b}}{2t}$$

where α is the angle of the cone, $w_{t}$ denotes the width of the upper kerf, $w_{b}$ is the width of the lower kerf and the thickness of the samples is shown by *t*. To specify the FPP length, an acrylic sheet positioned 80 degrees to the laser beams should be placed. Due to the effect of the beam on the sheet, the position of the FPP of the laser is determined (Figure 4a,b). Figure 4c shows the determination of the FPP using a CO_2_ laser.

Three input parameters including laser cutting speed, laser FPP and laser power of the laser are selected as input process parameters. Similarly, the FPP is located at the top or bottom of the workpiece, with a positive and negative FPP. In Figure 1, the FPP of the positive, zero and negative laser is shown from the left to right, respectively.

**Figure 5:**
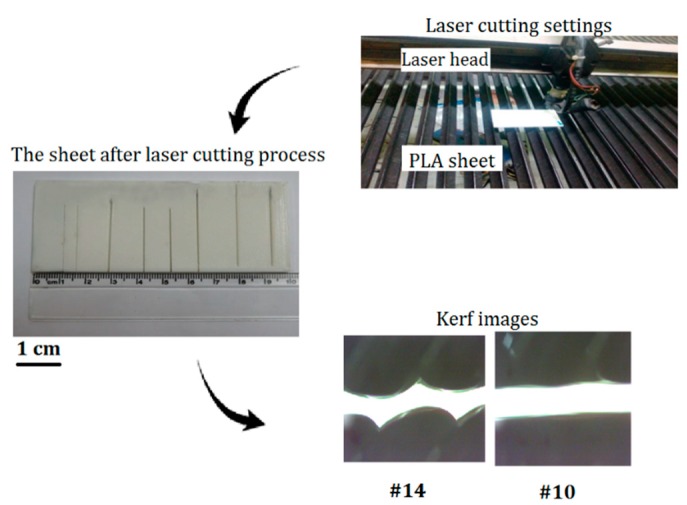
Laser cutting process and quality of some cutting kerf and surface.

By performing a few preliminary tests, changing the parameters and keeping other parameters constant, the range of parameters is determined. The cutting speed is changed from 4 to 20 mm/s in the first experiments. The FPP of −1.8 mm and the laser power of 40 W are considered in these experiments. Due to the fact that the material thickness used in this paper is 3.2 mm, the experiments are focused on the initial position of the FPP of 1.8 mm. A speed of 12 mm/s was selected as an appropriate speed with respect to the cut-off and the completeness of the cutting, while 40 W is selected as the best suited laser power in these experiments. The results indicated that when the FPP is located in zero position (exactly on the workpiece), the cutting quality is better than other cuts (Figure 5).

**Figure 6:**
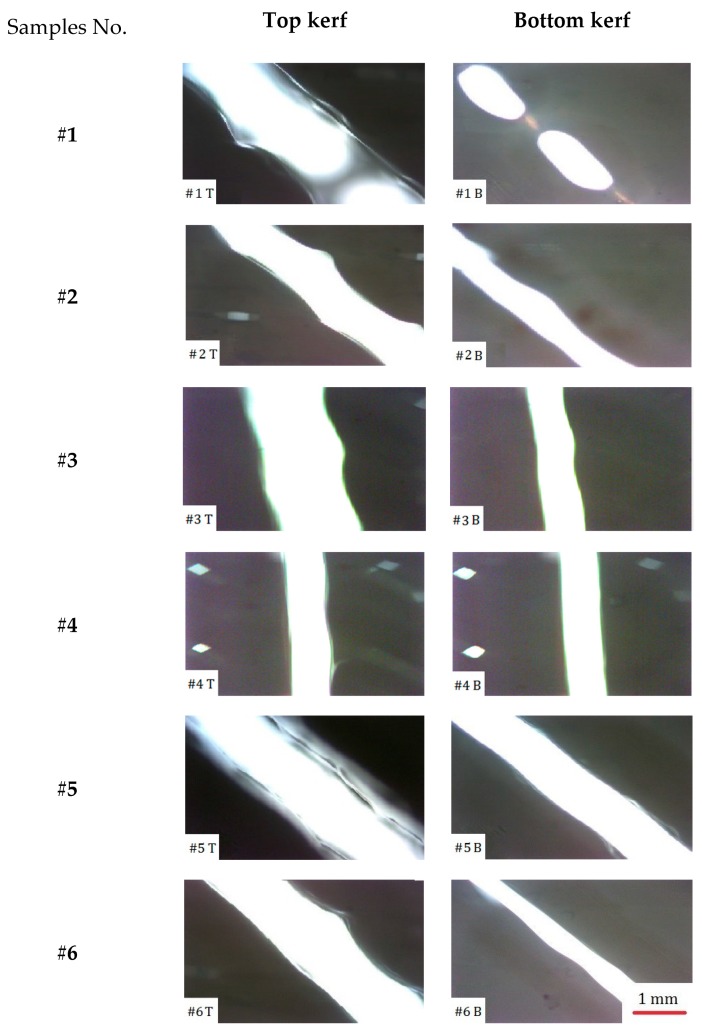
Cutting kerf feature of tests #1–6.

After photography, images are taken to obtain the upper and lower kerf and the tapering of each kerf is measured by the ImageJ software. Using this software, the geometric properties of the kerfs can be achieved. Figure 6 illustrates the top and bottom cutting kerfs of tests # 1–6.

## 4. Results and Discussion

The process scope is determined by performing a series of first tests, changing the parameters and keeping other parameters constant. To reach the complete cutting with proper appearance and non-defect on the parts, input and output parameters of this study were evaluated by RSM. In the following, each one of the output results for geometrical specifications are investigated.

### 4.1. Top Kerf Width

An analysis of variance for the top kerf width is shown in Table 5. All main parameters (FPP, laser power and cutting speed) are effective on top kerf width. Additionally, FPP^2^ and S^2^ are recognized as effective quadratic terms. According to the top kerf’s ANOVA table, interaction of two parameters is understandable. This means that the interaction of the FPP and cutting speed in ANOVA table for top kerf width (FPP×S) are effective terms. Based on actual and coded quantities, Equations (4) and (5) are presented.

(Upper Kerf)^1.36^ = 2368.68936 + 282.66295 × S − 1323.89295 × FPP + 102.75676 S × FPP − 20.32840 × S^2^ + 578.38346 × FPP^2^ (4)

(Upper Kerf)^1.36^ = 3351.24 − 11.61 × S − 1934.71 × FPP + 1972.93 × S × FPP − 731.82 × S^2^ + 5922.65 × FPP^2^ (5)

**Figure 7:**
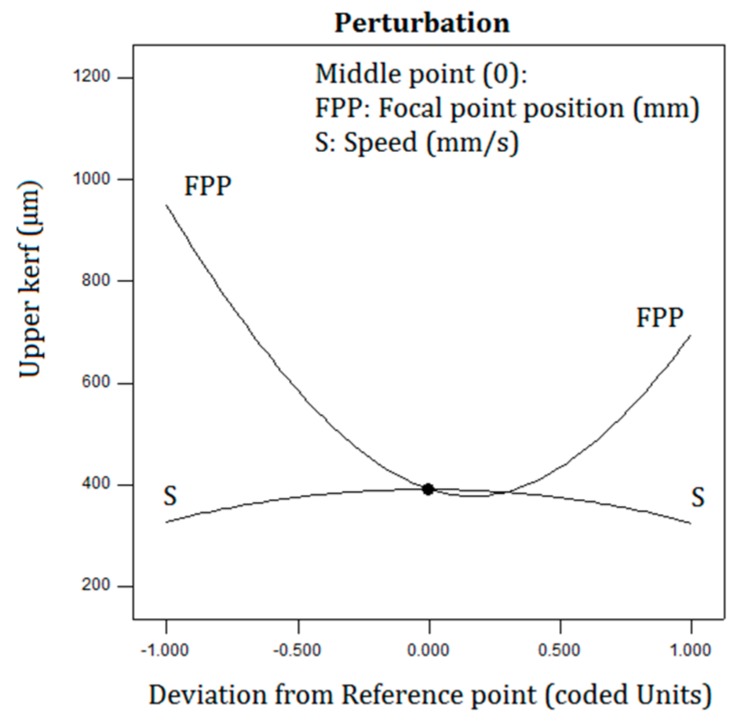
The top kerf width perturbation plot.

R-squared is the amount of the experimental data coverage which is obtained by the regression Equations (4) and (5). Figure 7 illustrates the top kerf width perturbation plot. The effect of the input parameters at the center point of the space from the design is compared by the perturbation plot. The perturbation of the top kerf width is illustrated with developing only single parameters over owned limited area, while other parameters are preserved fixed.

**Figure 8:**
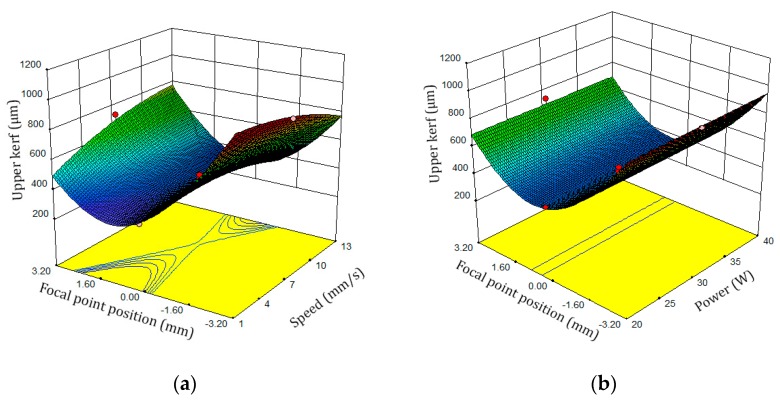
Response surface plots of the top kerf width. (**a**) FPP and cutting speed, (**b**) FPP and laser power

Figure 8 shows the response of the top kerf width surface plots. When the laser beam is closer to the surface, the FPP decreases, the case beam zone becomes tinier and the density of the laser beam increases. As shown in Figure 8a, by reducing the FPP and the cutting speed, the level of absorption energy is increased to the top of the sample surface and the top kerf width increases. Additionally, the top kerf width increases with reduction of the FPP and the increase of the laser power as shows in Figure 8b. The energy, which is radiated to the top kerf width increases, and consequently the top kerf width increases. By reducing the cutting speed, the interaction time of the beam radiated to the surface of the sample is increased, thus the top kerf width increases. This phenomenon can be argued with the heat input, and Equation (6) describes the amount of heat input based on the scanning speed and the power of the laser [51]:

(6) Heat input(J) = Laser power (W)/Scanning Speed (mm/s)

As shown in Table 2, in tests #12 and #15 (30 W power and zero position of FPP), only the cutting speed varies. The quality of the kerf is better (cutting speed was 1 mm/s), because the interaction of the laser beam with the PLA sheet increases at a slow rate, resulting in more heat absorption of the workpiece. Since the samples are produced by the FDM 3D printing technology, they are layered structures. When the cutting speed is low, the heat is absorbed more by the workpiece, which eliminates the roughness. It is worthwhile to mention that, by increasing the cutting speed, the interaction of the laser action with the workpiece decreases and the heat is less absorbed into the workpiece, affecting the top kerf width. With increasing cutting speed, the kerf quality does not look good, and according to the images taken by the optical microscope, the roughness of the kerf surface is high.

### 4.2. Bottom Kerf Width

It is clear from Table 6 that the FPP and cutting speed are effective terms for main parameters on bottom kerf width, while several quadratic terms are of significance for bottom kerf width (FPP^2^ and S^2^). Additionally, the interaction effect of FPP and cutting speed (FPP × S) has also been indicated as a significant term. According to ANOVA Table 6, Equations (7) and (8) represents the regression equation for the bottom kerf width based on the significant terms.

(Lower Kerf)^0.82^ = 106.47655 + 10.72785 × S − 32.99704 × FPP + 3.87539 × S × FPP-0.90775 × S^2^ + 5.37525 × FPP^2^ (7)

(Lower Kerf)^0.82^ = 137.09 − 11.88 × S − 18.78 × FPP + 74.41× FPP × C − 32.68 × S^2^ + 55.04 × FPP^2^ (8)

**Figure 9:**
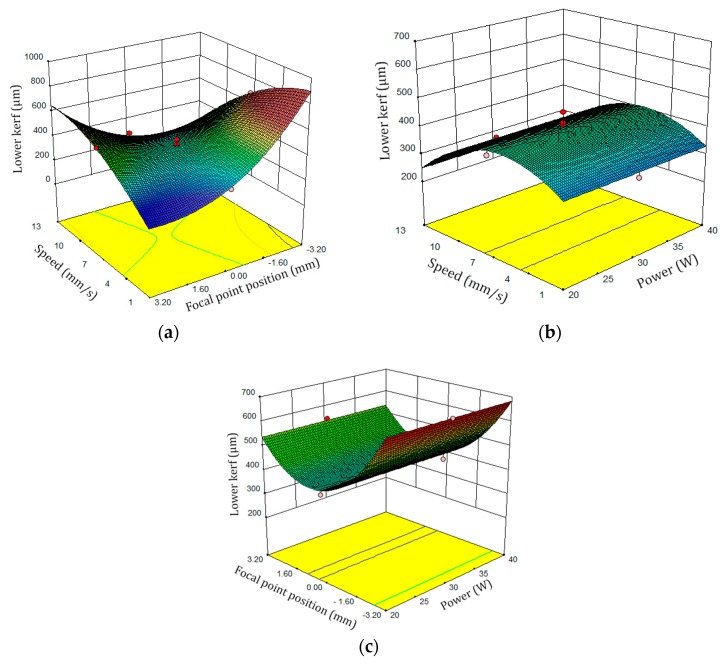
Bottom kerf width surface plot: (**a**) scanning speed and FPP, (**b**) laser power and cutting speed, (**c**) FPP and laser power.

**Figure 10:**
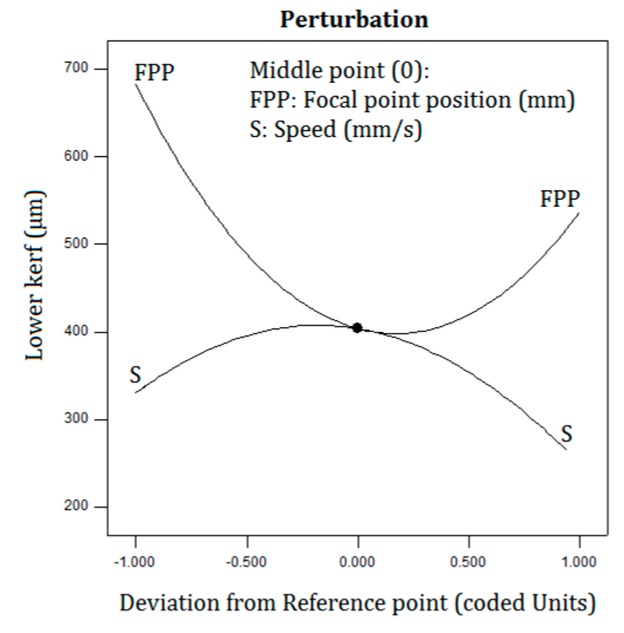
Perturbation plot of bottom kerf width.

Surface plot of the bottom kerf width is shown in Figure 9. Based on Figure 9, when the laser cutting speed and the FPP parameters are increased, the laser beam interaction effect on the PLA sheet is low and this phenomenon makes the bottom kerf width samples have low amounts. Additionally, In Figure 10, the perturbation plot of the bottom kerf width is illustrated. Since the FPP parameter in the regression Equations (6) and (7) and its F-Value in ANOVA table are greater than the cutting speed factors, the slope of the FPP curve is greater than the other curves. Therefore, FPP parameter has the greatest effect on the bottom kerf width.

### 4.3. Ratio of the Top Kerf to Bottom Kerf

In Table 7, a variance analysis for the ratio of the top kerf to bottom kerf is presented. In this table, FPP and cutting speed are effective linear parameters. Additionally, FPP^2^ and S^2^ are quadratic terms which have significant effects. The scanning speed and the FPP interaction effect (S × FPP) are the only significant interaction. Regression equations for the ratio of top to bottom kerf are presented in Equations (9) and (10).

(Ratio)^−0.09^ = 0.99379 + 0.00376236 × S − 0.00622428 × FPP + 0.00136254 × S × FPP − 0.000403990 × S^2^ + 0.00277260 × FPP^2^ (9)

(Ratio)^−0.09^ = 1 − 0.011 × B + 0.011 × C + 0.026 × B × C − 0.015 × B^2^ − 0.028 × C^2^ (10)

**Figure 11:**
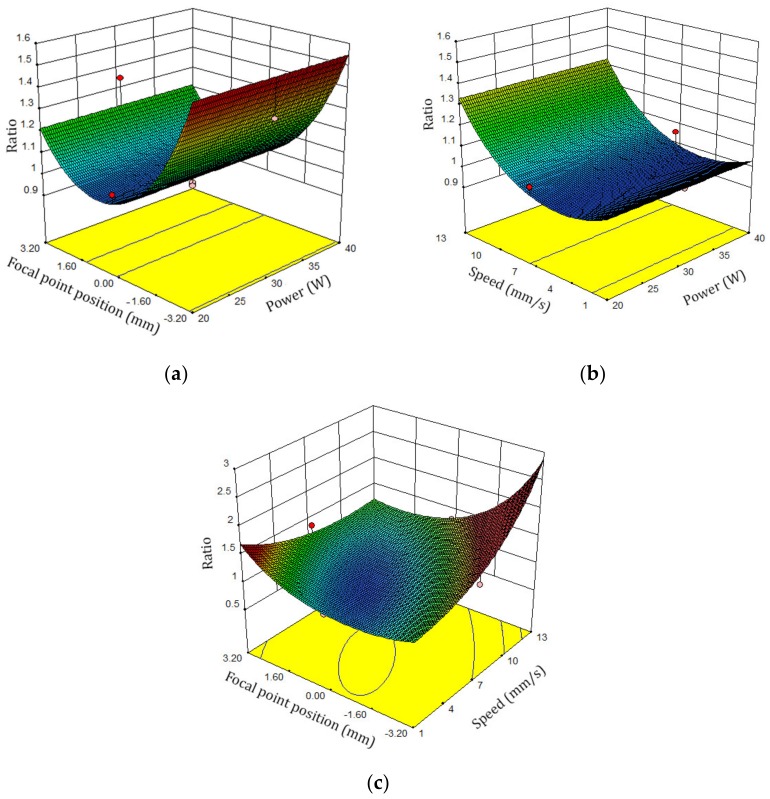
Ratio of top kerf to bottom kerf surface plots: (**a**) FPP and laser cutting speed, (**b**) laser power and laser cutting speed, (**c**) FPP and cutting speed

**Figure 12:**
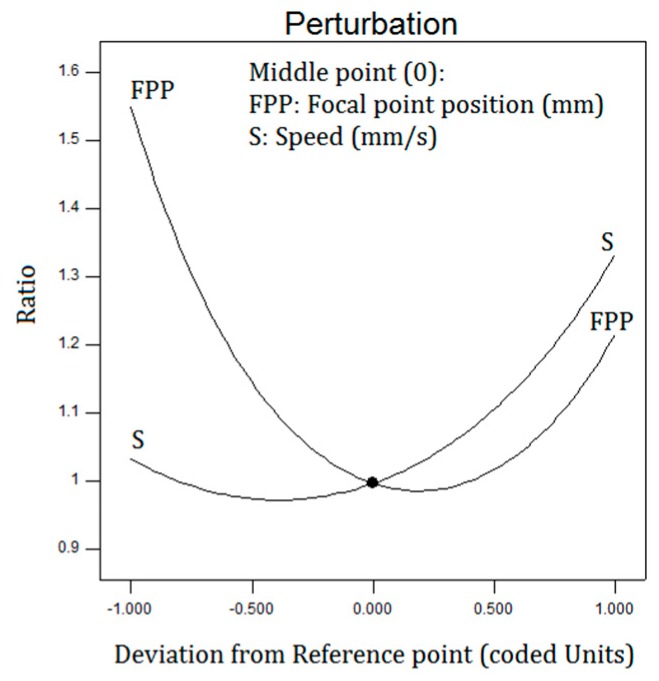
Perturbation plot of the ratio of top kerf to bottom kerf.

In Figure 11, the response surface plots for the top to bottom kerf is demonstrated. According to the FPP, cutting speed, and laser power, by increasing the scanning speed, the ratio of the top to bottom kerf increases, see Figure 11a,c. Figure 12 shows the ratio of top to bottom kerf perturbation plot.

### 4.4. Taper

Taper ANOVA is listed in Table 8. S and FPP are effective linear parameters. Additionally, FPP^2^ is the quadratic term and has a significant effect. Taper regression equations based on coded values are presented in Equations (11) and (12).

(Taper + 0/50)^1.02^ = 0.072007 + 0.070500 × S + 0.17490 × FPP − 0.054374 × S × FPP + 0.18896 × FPP^2^ (11)

(Taper + 0/50)^1.02^ = 0.57 + 0.42 × S − 0.66 × FPP − 1.04 × S× FPP + 1.93 × FPP^2^ (12)

**Figure 13:**
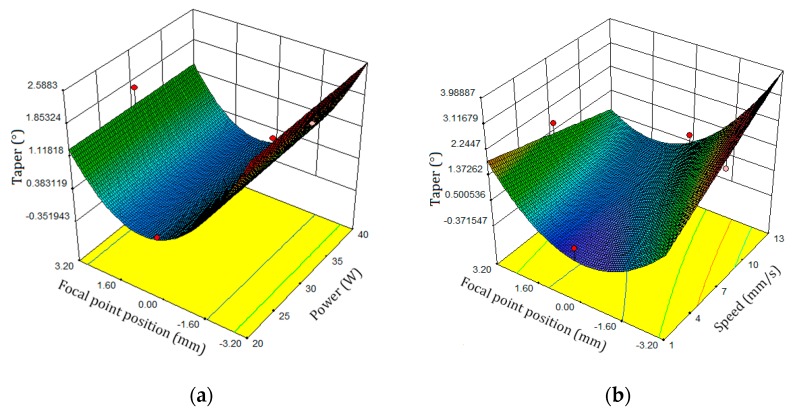
Taper response surface plate: (**a**) FPP and Power, (**b**) FPP and laser cutting speed.

**Figure 14:**
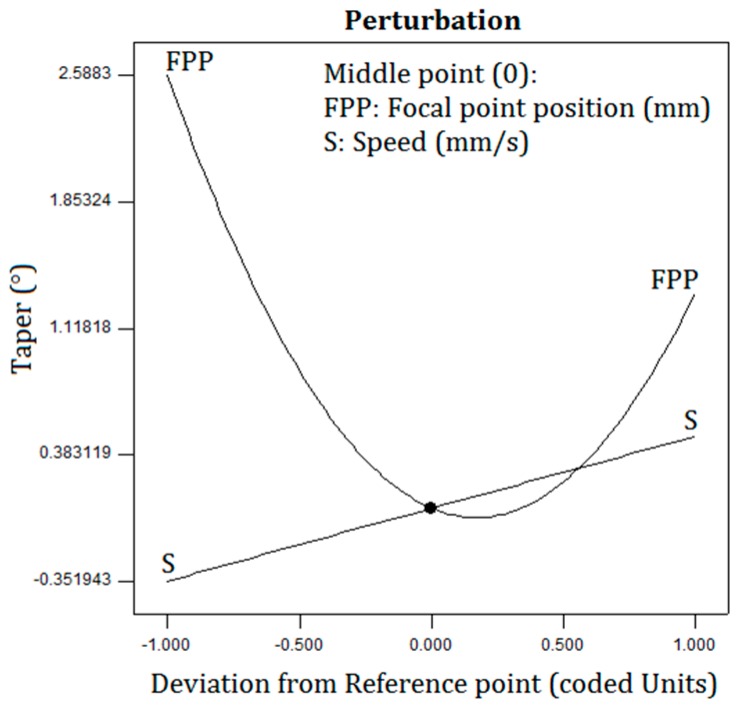
Perturbation plot of the taper.

Figure 13a shows the effect of the position parameters of the laser power and the FPP on the taper. The taper is increased by changing the FPP. The parameters’ effect of the laser cutting speed and FPP on the taper is shown in Figure 13b. While the laser cutting speed and FPP parameters are increased, the taper increases. As can be concluded, the greatest taper occurs at the major FPP and highest laser cutting speed. The effect of the FPP and laser cutting speed on the taper is shown in Figure 14. As indicated in the diagram, the taper is linearly decreasing while the laser cutting speed decreases, and the taper increases as the position of the FPP decreases.

## 5. Optimization

**Figure 15:**
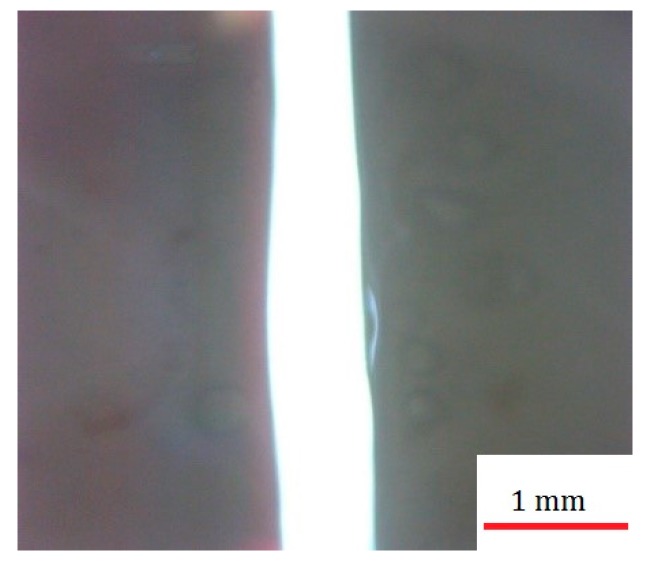
Image of the cut kerf of the optimized sample #5.

In this section, some tests are investigated to validate the quantities of parameters and determine the percentage of possible error of the difference in the output responses of the statistical method and the laboratory method [26,52]. Table 9 shows the actual, predicted and error rate of the experimental method and the experimental design method for the output responses. As shown in Table 9, the error rate of experimental and optimization methods is below 15% for the output results of geometric characteristics (the top and bottom kerfs, the ratio of the top and bottom kerf incisions of optimization samples) and this is an acceptable error rate for this study. The best test is shown for optimum setting in Figure 15. The walls of the best optimum setting are in superb condition. Additionally, around the edges of the laser cutting routs, no defects appear and samples are very suitable for use.

## 6. Conclusions

The post-processing of PLA sheets fabricated by FDM 3D printing was investigated, by implementing CO_2_ laser cutting. The effects of laser cutting process parameters on the geometrical dimension of the kerf (e.g., bottom and top kerf width, ratio of the top kerf to bottom kerf, taper) were studied by the response surface method. The following conclusions could be drawn from the experimental study:

(1) Dimensional accuracy of the FDM 3D-printed PLA parts can be improved by laser cutting as a post processing step. The laser can cut the samples easily, whereas the kerfs dimension quality has acceptable features.
(2) Decreasing the FPP range from zero to −3 mm causes a decline in the top and bottom kerf width but decreasing more than −3 mm has an inverse effect.
(3) Kerf taper is increased by changing the FPP. It should be mentioned that the laser cutting speed and FPP in the liner terms based on the ANOVA table of kerf taper has effective influence on kerf Taper amount.
(4) The best optimum sample is achieved with 1.19 mm/s cutting speed, 36.49 W power and 0.53 mm. Focal plane position input parameters have good physical features after the laser cutting process when 276.9 μm top and 261.5 μm bottom kerf width is cut by laser.
(5) The overall conclusion is that by locating the laser spot point in the profundity of the sheet, the laser cutting process results in the best quality.

## Figures and Tables

**Table 1:** Three levels of cutting parameters.

Variable	Symbol	Unit	−2	−1	0	+1	+2
Scanning speed	S	mm/s	1	4	7	10	13
Laser power	P	W	20	25	30	35	40
Focal plane position	FPP	mm	−3.2	−1.6	0	1.6	3.2

**Table 2:** Results overview for laser cutting experiments.

Sample No.	Input Variables			Output Responses			
	P (W)	S (mm/s)	FPP (mm)	Top Kerf Width (μm)	Bottom Kerf Width (μm)	Ratio	Taper (°)
1	35	4	−1.6	631.30	576.950	1.0942	0.4865
2	30	13	0	358.62	273.490	1.311	0.7620
3	30	7	0	387.93	406.890	0.953	−0.1697
4	30	7	0	400	415.517	0.962	−0.1342
5	30	7	−3.2	934.60	681.230	1.372	2.267
6	30	7	3.2	732.75	543.670	1.347	1.692
7	25	10	1.6	413.79	386.630	1.070	0.2431
8	25	10	−1.6	472.41	332.720	1.419	1.250
9	25	4	1.6	332.75	303.500	1.096	0.2618
10	25	4	−1.6	608.62	546.670	1.113	0.5545
11	35	10	−1.6	582.75	383.390	1.519	1.784
12	30	7	0	429.31	453.870	0.945	−0.2198
13	40	7	0	385.34	364.780	1.056	0.1840
14	35	10	1.6	429.31	453.870	0.945	−0.2198
15	30	1	0	320.04	310.210	1.031	0.0880
16	20	7	0	401.72	387.500	1.036	0.1273
17	35	4	1.6	381.03	403.440	0.944	−0.2006

**Table 3:** Printer specifications.

Device Parameters	Parameter Range
Type of printer	FDM Sizan Model 3
Print size	20 × 20 × 20 cm
Laying accuracy	30 μm
Temperature of plate	110 °C
Nozzle diameter	0.5 mm
Temperature of nozzle	260 °C
Speed of printer	300 mm/s

**Table 4:** Specification of polylactic acid (PLA).

Feature	Amount
Name	Polylactic acid (PLA)
Crystallinity	37%
Chemical formula	(C3H4O2)n
Tensile modulus	2.7–16 GPa
Density	1.210–1.430 g·cm^−3^
Melting point	150 to 160 °C (302 to 320 °F)
Glass transition	60–65 °C
Injection mold temperature	178 to 240 °C (353 to 464 °F)

**Table 5:** Revised ANOVA of top kerf width.

Source	Sum of Squares	Degree of Freedom	Mean Square	F-Value	*p*-Value
Model	76,260,000	5	15,250,000	65.71	<0.0001
S	539	1	539	0.002322	0.9624
FPP	14,970,000	1	14,970,000	64.50	<0.0001
S × FPP	1,946,000	1	1,946,000	8.38	0.0146
S^2^	768,000	1	768,000	3.31	0.0962
FPP^2^	50,300,000	1	5,030,000	216.70	<0.0001
Residual	2,553,000	11	232,100		
Lack of Fit	2,426,000	9	269,500	4.23	0.2057
Pure Error	127,300	2	63,673.87		
Total	78,810,000	16			
R-squared = 96.76%			R-squared (Adj) = 95.29%		

**Table 6:** ANOVA table for bottom kerf width.

Source	Sum of Squares	Degree of Freedom	Mean Square	F-Value	*p*-Value
Model	12,718.65	5	2543.73	22.69	<0.0001
S	564.87	1	564.87	5.04	0.0463
FPP	1411.01	1	1411.01	12.59	0.0046
S× FPP	2768.24	1	2768.24	24.69	0.0004
S^2^	1531.34	1	1531.34	13.66	0.0035
FPP^2^	4344.45	1	4344.45	38.75	0.0001
Residual	1233.26	11	112.11		
Lack of fit	1138.58	9	126.51	2.67	0.3019 not
Pure error	94.68	2	47.34		
Total	13,951.91	16			
R-Squared = 91.16%			R-Squared (Adj) = 87.14%		

**Table 7:** ANOVA table of the ratio of top to bottom kerf.

Source	Sum of Squares	Degree of Freedom	Mean Square	F-Value	*p*-Value
Model	0.002530	5	0.0005060	8.52	0.0016
S	0.0005163	1	0.0005163	8.69	0.0132
FPP	0.0004497	1	0.0004497	7.57	0.0188
S × FPP	0.0003422	1	0.0003422	5.76	0.0352
S^2^	0.0003033	1	0.0003033	5.11	0.0451
FPP^2^	0.001156	1	0.001156	19.47	0.001
Residual	0.0006532	11	0.00005938		
Lack of Fit	0.0006519	9	0.00007243	111.44	0.0089
Pure Error	0.0000013	2	0.0000006499		
Total	0.003183	16			
R-Squared = 79.48%			R-Squared (Adj) = 70.16%		

**Table 8:** Taper analysis ANOVA.

Source	Sum of Squares	Degree of Freedom	Mean Square	F-Value	*p*-Value
Model	8.83	4	2.21	19.18	<0.0001
S	0.72	1	0.72	6.22	0.0282
FPP	1.73	1	1.73	15.06	0.0022
S × P	0.54	1	0.54	4.74	0.0502
FPP^2^	5.84	1	5.84	50.72	<0.0001
Residual	1.38	12	0.12		
Lack of fit	1.38	10	0.14	70.47	0.0141
Pure error	0.003908	2	0.001954		
Total	10.21	16			
R-Squared = 86.48%			R-Squared (Adj) = 81.97%		

**Table 9:** Input and output parameters for optimum settings.

Solution	Input Parameters Optimum				Output Results		
1	**S (mm/s)**	**P (W)**	**FPP (mm)**		**Top Kerf (μm)**	**Bottom Kerf (μm)**	**Ratio**
	1.4	30.18	0.53	Actual	327.07	307.69	1.052
				Predicted	287.056	289.735	0.945
				Error%	13.9	6.19	11.32
2	7.97	24.27	0.98	Actual	406	392	1.035
				Predicted	394.29	404.895	0.97
				Error%	2.96	−3.18	6.7
3	3.04	27.64	0.45	Actual	387.6	370	1.047
				Predicted	333.659	351.065	0.95
				Error%	16.1	5.9	10.21
4	2.42	36.57	0.47	Actual	400	364.6	1.09
				Predicted	320.037	333.159	0.952
				Error%	24.8	9.4	14.4
5	1.19	36.49	0.53	Actual	276.9	261.5	1.05
				Predicted	287.077	289.752	0.945
				Error%	−3.5	−9.7	11.11
